# Multiagent Q-Learning-Based Mobility Management for Multi-Connectivity in mmWAVE Cellular Systems

**DOI:** 10.3390/s23177661

**Published:** 2023-09-04

**Authors:** Si A Ryu, Duk Kyung Kim

**Affiliations:** Department of Information and Communication Engineering, Inha University, Inchon 22212, Republic of Korea; ryusia98@daum.net

**Keywords:** multiagent, distributed Q learning, mobility management, multi-connectivity, mmWAVE

## Abstract

Effective mobility management is crucial for efficient operation of next-generation cellular systems in the millimeter wave (mmWave) band. Massive multiple-input–multiple-output (MIMO) systems are seen as necessary to overcome the significant path losses in this band, but the highly directional beam makes the channels more susceptible to radio link failures due to blockages. To meet stringent capacity and reliability requirements, multi-connectivity has attracted significant attention. This paper proposes a multiagent distributed Q learning-based mobility management scheme for multi-connectivity in mmWave cellular systems. A hierarchical structure is adopted to address the model complexity and speed up the learning process. The performance is assessed using a realistic measurement data set collected from Wireless Insite in an urban area and compared with independent Q learning and a heuristic scheme in terms of handover probability and spectral efficiency.

## 1. Introduction

As the demand for mobile traffic and data rates skyrocket, the use of millimeter wave (mmWave) and terahertz (THz) bands is becoming increasingly necessary due to the shortage of available bands in the sub-6 GHz band [1]. However, the high carrier frequencies of these bands result in significant path losses, which limits their transmission range. To overcome this challenge, the 5G new radio (NR) network can adopt an increased cell density and multiple narrow beams with a massive multiple-input–multiple-output (MIMO) system [2]. However, the use of a narrow beam reduces the service area of a single beam, making the link more susceptible to radio link failures (RLFs) due to blockages. Therefore, it is challenging to ensure consistent signal quality in urban environments, where blockages caused by buildings or obstacles occur frequently [3].

Effective mobility management is crucial for mobile systems, which can be impacted by environmental dynamics such as user equipment (UE) movement and blockages. Multi-connectivity (MC) has recently emerged as a promising approach to enhance reliability and prevent frequent handovers (HOs) [4]. MC refers to the simultaneous connection of multiple independent communication links to a single UE. By combining the signals from all connected links at the receiver, throughput can be increased, and reliability can be improved by maintaining connections with extra links.

The conventional HO method used for selecting the optimal BSs and beams requires a measurement and comparison of the reference signal receiving powers (RSRPs) of the serving and neighboring BSs/beams, inducing signaling overheads and processing delays. As a massive MIMO system is employed and cells are deployed more densely in a 5G NR system, it is challenging to optimize the HO process by simultaneously considering several complex requirements, e.g., minimizing the signaling overhead, maximizing the user experience, and reducing the network burden. Moreover, in practical scenarios, such as in urban areas, the surrounding environment is quite different and changes as the UE moves, and its characteristics cannot be captured appropriately using mathematical models. In [5], HO-related issues are addressed within the context of heterogeneous networks (HetNets). Particularly in high-speed 5G environments, the imperative task is to determine the optimal configuration of handover control parameters in mobility management. In pursuit of this objective, this study presented HO techniques grounded in weight functions, fuzzy logic schemes, and machine learning along with relevant prior research endeavors. In [6], a method is proposed that concurrently refines time-to-trigger (TTT) and handover margins (HOMs) based on a fuzzy logic approach. Utilizing the input parameters of RSRPs, reference signal received quality (RSRQ), and UE rates, the simultaneous adjustment of TTT and HOMs resulted in enhanced performance when compared with competitive algorithms. In addition to the fuzzy logic in particular, the ML framework is considered an appropriate tool for mobility management; here, an ML model or policy can be trained offline in advance, and the trained model can be used in real time.

Multiagent frameworks are generally classified as either distributed or centralized approaches. In the centralized approach, a central controller or a single agent collects observations from all agents and takes actions for all of them, forwarding the selected action information to each agent. In contrast, the distributed approach involves each agent independently selecting actions based on its own policy. The centralized approach can be suitable for selecting globally optimal actions, as it considers information from all agents at once, but it is more complex than the distributed approach. The complexity of the distributed approach is related to the size of the Q table for each agent, while the complexity of the centralized approach increases exponentially with the number of agents and the size of their Q tables since all information is gathered and learned by the central agent.

To overcome these problems pertaining to centralized algorithms, we propose a multiagent distributed Q learning (DMAQ) approach for mobility management in massive MIMO mmWave cellular systems. Because distributed Q learning does not require a deep neural network for function approximation, the proposed algorithm can be utilized in practical UEs with limited computation power. To reduce the model complexity and improve learning speed, we adopt a hierarchical structure. We obtain a realistic measurement dataset using a ray-tracing tool (Wireless Insite) that employs the geographic/map information of Cheongna-dong, Inchon, Korea, an urban area. The effectiveness of the proposed scheme is verified via intensive simulations and compared with independent Q learning and heuristic algorithms in terms of handover probability (HOP) and spectral efficiency (SE).

The contribution of this work can be summarized as follows.

- The multiagent distributed Q learning-based mobility management scheme is proposed to efficiently handle the MC in mmWave cellular systems, in which the channels become more susceptible to RLFs owing to the highly directional beams. When multiple UEs are serviced simultaneously, in addition to the signal gain from multiple connections, the possible interference with other UEs should be avoided to maximize overall system performance. The proposed scheme can be a good solution for this situation.
- Owing to the massive MIMO system deployment in mmWave bands, a large number of narrow beams are provided to service overall cell coverage. This introduces a complexity and convergence problem for RL-based mobility management, as it must simultaneously search for the best beam and BS. To mitigate this problem, we adopt a hierarchical structure, i.e., a BS level and beam level. The optimal candidate beams are determined on a BS basis at the beam level. Following this, the multiple connections can be chosen only from the set of recommended candidate beams, rather than among the beams of all the BSs in the service area. This can considerably reduce the dimensions of the RL model and accelerate the convergence speed.
- Multiple UEs are serviced simultaneously, where a beam/BS favorable to a particular UE can cause serious interference with other UEs even though they are spatially separated. Instead of a centralized model requiring a large volume of information exchange among the BSs, a distributed model is adopted in our model. To achieve globally optimal performance, we propose sharing of the local rewards of the agents. Using this reward sharing (RS), the performance can be further improved relative to that of the MAQ model without RS.

The rest of the paper is organized as follows. We review related works in Section 2. Our system model is explained in Section 3 and the heuristic mobility management scheme is introduced in Section 4. We propose a multiagent distributed Q learning-based mobility management scheme in Section 5. Then, the simulation setups are explained, and the performance results are analyzed in Section 6. Finally, we conclude our work along with future directions in Section 7.

## 2. Related Works

### 2.1. Mobility Management with Multi-Connectivity

Recently, as wider bandwidths have become accessible, the utilization of high-frequency bands, such as the mmWave and THz bands, has attracted considerable interest. However, the use of these bands leads to reduced coverage owing to large path losses [7]. In 5G NR networks, a massive MIMO technique has been introduced. Herein, as the number of antenna elements increases, the coverage area can be expanded by compensating for the path losses with a high antenna gain. However, as the beam becomes narrower, the cell coverage comprises more beams. The link becomes more vulnerable to blockages, and this can lead to sudden signal changes, resulting in frequent HOs. In particular, frequent HOs cause packet loss or delay, thereby increasing the possibility of RLFs. Therefore, an appropriate mobility management scheme is required for allocating the appropriate beams/BSs.

To enhance the HO robustness in mmWave communications, a prediction-based HO scheme is proposed. Unlike conventional reactive schemes, this type of proactive scheme encompasses a prediction of whether each link is line-of-sight (LoS) or non-LoS (NLoS) in the next move. For this prediction, camera and sensor information is used in addition to the radio frequency signal measurements [8]. However, this technique has limited applications, such as for straight roads. A Markov chain model was developed in [9], where each link could be in one of the following three states: outage, LoS, and NLoS. The state transition probabilities were derived based on the distance between the UE and BS with the assumption of a random distribution of the UEs and BSs. The optimal cell association was obtained by using a value iteration algorithm to maximize the reward. In addition, Ref. [10] addressed the contradiction between optimizing mobility robustness and load distribution in a 4G/5G coexistence scenario. The proposed method establishes HO decisions by factoring in RSRPs and user speed, leading to a notable reduction in HO ping-pong probability and HO failure rate. However, these only considered a single connection, and the interference from other cells was not incorporated in the rate calculation.

Recently, MC has been studied for mobility management in the mmWave and THz bands. In [11], the authors developed a Markov chain model for mathematically analyzing the HO operations in MC, where the BSs and blockers were randomly placed. The link status between a BS and the UE could exist in one of the following four states: undiscovered/blocked, discovered, candidate, and associated. The state transition probabilities were derived by considering the blockage duration, HO execution, and BS discovery time. The authors analyzed the out-of-service and RLF probabilities and claimed that MC was effective in providing low delays and high reliabilities. In [12], the authors proposed two access point association approaches and evaluated their performance based on a Markov chain model. The connection was considered successful when at least one link had LoS status, which could be improved by associating multiple links. A single UE was considered, and the LoS probability was derived from the assumption of randomly placed dynamic blockages as well as self-blockages.

As such, MC has been studied primarily based on mathematical models in the mmWave and THz bands. However, an in-depth investigation of mobility management in real urban environments has yet to be conducted, particularly for MC. In addition, only a single UE has been considered in the mobility analyses. However, to increase the SE, multiple UEs can be scheduled simultaneously with the same frequency resources across multiple BSs. The interference from neighboring BSs serving other UEs should be considered to maximize the system-wide performance. This involves cooperation between the BSs and necessitates efficient multiuser mobility management with MC using massive MIMO systems in the mmWave and THz bands.

### 2.2. RL in Mobility Management

**Figure 1:**
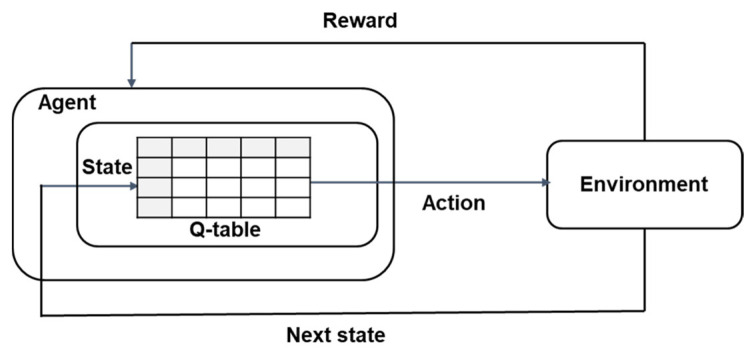
Diagram of a Q learning framework.

The RL model comprises an agent, state, action, and reward as depicted in Figure 1. Each agent has a Q table that saves the Q value for all the existing state–action pairs. The Q value is calculated when an action is taken in a certain state; the higher the value, the better it is to select the corresponding action. The Bellman equation is used to update the Q value, as follows [13]:

(1) $$Q\left(s,a\right)=Q\left(s,a\right)+\alpha \left(r\left(s,a\right)+\gamma \underset{a^{\prime }}{\max}Q\left(s^{\prime },a^{\prime }\right)-Q\left(s,a\right)\right).$$

Q(s,a) is the Q value when action a is taken in the current state s; $r\left(s,a\right)$ is the reward value; and $Q(s^{\prime },a^{\prime })$ is the value when the predicted action $a^{\prime }$ is taken in the updated next state $s^{\prime }$. According to these definitions, $\max Q\left(s^{\prime },a^{\prime }\right)$ is the largest Q value that can be obtained in the next state, that is, the Q value when the most optimal action is selected in a certain state. Further, α is the learning rate, and it determines the training convergence speed. γ is the discount rate, and it represents confidence in future rewards. For example, if γ is zero, the Q value is calculated without considering future rewards. For successful convergence in RL, the concepts of exploration and exploitation are used. Among these, exploration involves trying new actions to allow the agent to expand its knowledge of the evaluation to obtain long-term benefits, whereas exploitation involves choosing an action based on the learned Q values or policies. In the initial stage of the training, exploration should be conducted on a trial-and-error basis, and the proportion of exploitation should be increased as the training progresses.

The objective of the Q learning model is to determine the best action for the current state. An agent tries an action at a particular state, evaluates the reward, and moves to the next state based on the selected action. The Q table is updated according to (1). By repeatedly trying actions and updating rewards, the agent learns to select the optimal action based on a long-term discounted reward. The agent determines the optimal action with the maximum expected reward value. If more than one action has the same maximum Q value, one action is taken at random. Because all Q values in the Q table are initialized to zero, random action is selected at the beginning of learning.

Recently, there have been several studies exploring the combination of mobility management and ML to improve mobility management [14,15,16,17,18,19,20,21,22]. The 3rd Generation Partnership Project (3GPP) has also been working on the integration of 5G and artificial intelligence (AI)/ML to meet the service requirements of 5G NR, which include ultrareliable low-latency communications and enhanced mobile broadband [14]. There are three main types of ML: supervised, unsupervised, and reinforcement learning (RL). Among these, supervised learning and unsupervised learning are used for classifying or predicting data based on labeled or unlabeled datasets, respectively. For example, in [15], supervised learning was combined with HO operations in a 5G NR network to propose a proactive mobility decision-making method. The authors split the ML model into a cell-level model for selecting a serving base station (BS) and a beam-level model for selecting the serving beam for the BS based on the UE’s present location and predicted RSRP at the next location. In [16], the authors proposed an intelligent dual connectivity mechanism for mobility management, which predicted the mobility pattern of a UE based on its historical position data and long short-term memory (LSTM) architecture. The network made an HO decision based on the predicted mobility pattern of the UE. However, these studies only considered single-user scenarios. In [17], the authors provided a survey of HO management and discussed the use of the ML framework in mobility management. They claimed that RL is an effective method, especially for ultra-dense small-cell 5G networks, and highlighted the importance of effectively managing inter-cell interference to increase network capacity in the presence of multiple UEs. In [18], the authors proposed a path skeleton that defines each path based on its angle of arrival (AoA), angle of departure (AoD), and channel gain. This approach allows for faster beamforming compared to considering all possible beams, which can involve high overhead, particularly in massive MIMO systems. They also introduced a Q learning-based HO scheme to select the best backup BS for a single UE moving along a predetermined route, with the aim of maximizing the trajectory rate. Another study [19] used a double deep-*Q* network to improve the dual active protocol stack (DAPS) HO in terms of throughput and RLF for a heterogeneous deployment of macro and micro BSs, using the Madrid Grid model from the “METIS-II” project. However, both studies only considered a single UE and did not account for interference from other UEs. In [20], the authors proposed a solution for mitigating interference in 5G mmWave communications using beamforming and non-orthogonal multiple access (NOMA) to improve the network’s aggregated rate. They solved the problem of selecting the optimal number of beams and allocating users to those beams using transfer Q learning. In [21], a multiagent Q learning framework was proposed, featuring knowledge exchange to counterbalance the challenges posed by a network entity functioning in a distributed manner with only partial observations. Within this framework, individual agents ascertained whether to transmit during each time slot while factoring in inter-cell interference considerations. In [22], a distinctive approach optimized the positioning of each unmanned aerial vehicle (UAV) using multi-agent Q learning. The objective here was to maximize the cumulative rate across multiple UAVs. However, their approach did not consider mobility management or MC, and assumed a simple LoS link that may not reflect real-world environments.

## 3. System Model

**Figure 2:**
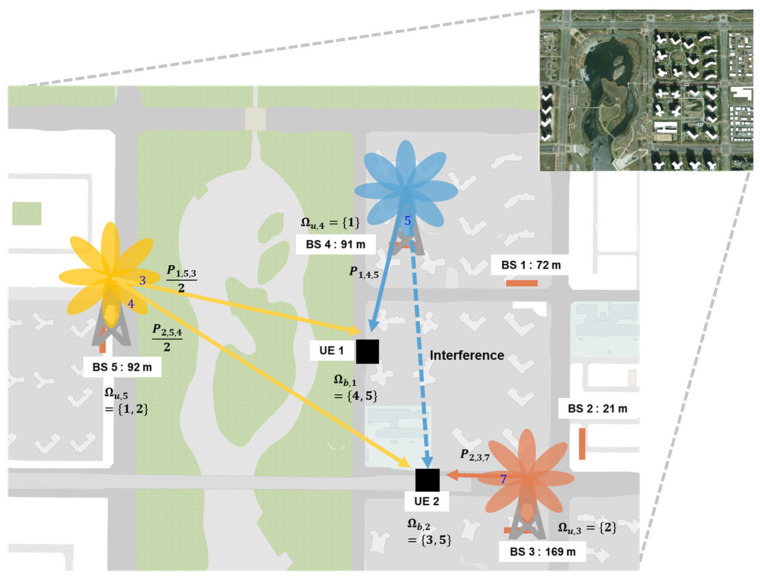
Typical urban scenario, Inchon, Korea (5 BSs, 2UEs).

As shown in Figure 2, Cheongna-dong, Inchon, Korea features high-rise apartment buildings and a large lake park, which can be considered a typical urban scenario. The BSs are installed at the tops of the buildings. The locations and heights of the 5 BSs are obtained from 5G site information provided by mobile network operators in Korea. The service area is approximately 1200 m × 850 m. An example case of the serving beam and interference beam is shown in the Figure 2, where there are two UEs. When considering the multiple UE case, interference from the beam selected by other UEs exists. For example, beam index 5 of BS4 serves UE1 and induces interference to UE2. Typically, a single BS may serve multiple UEs, such as BS5. The transmitted power of the BS is then divided by the number of serving beams.

We consider two categorized MC transmission modes: joint transmission (JT) and dynamic point selection (DPS). In JT mode, all multiple connected links are simultaneously used for transmission [23]. On the other hands, DPS selects only one link for transmission among multiple connected links, and the other connections remain ready for possible transmission. Notably, the interference from other beams may be larger in JT mode than in DPS mode. However, the UE can receive a stronger signal in the JT mode because this mode combines signals from multiple links.

We introduce a set of serving UE indices of the k-th BS $\Omega_{u,k}$. Following this, the actual beam power of the k-th BS is divided by $\left|\Omega_{u,k}\right|$. We let $\Omega_{b,i}$ be the index set of the serving BSs of the i-th UE. $\Omega_{t,i}$ is a set of transmitting BS(s) of the i-th UE which is used to transmit data. In JT mode, $\Omega_{b,i}$ equals $\Omega_{t,i}$ because all selected serving beams are used for transmitting while $\Omega_{t,i}\subset \Omega_{b,i}$, |$\Omega_{t,i}$| = 1 in DPS mode. $P_{i,k,f}$ is the received power of the i-th UE from beam f of the k-th BS. With these notations, the received power of UE i can be calculated as the sum of the received signal strengths from all serving BSs via the corresponding beam.

(2) $$P_{r,i}=\sum_{k\in \Omega_{t,i},\;f\in F_{k}}\frac{P_{i,k,f}}{\left|\Omega_{u,k}\right|}$$

The interference at UE i is the summation of the signal powers from the beams serving the other UEs.

(3) $$I_{i}=\sum_{n\neq i}\sum_{k\in \Omega_{t,\;n},f\in F_{k}}\frac{P_{i,k,f}}{\left|\Omega_{u,k}\right|}$$

Then, the received signal-to-interference-plus-noise ratio (SINR) of UE *i* can be obtained as follows:

(4) $$SINR_{i}=\frac{P_{r,\;i}}{I_{i}+\sigma^{2}}$$

For an example scenario in Figure 2, $\Omega_{b,1}=\left\{4,\;5\right\}$ and $\Omega_{b,2}=\left\{3,\;5\right\}$. Thus, we can obtain $\Omega_{u,3}=\left\{2\right\}$, $\Omega_{u,4}=\left\{1\right\}$, and $\Omega_{u,5}=\left\{1,\;2\right\}$. 

## 4. Heuristic Mobility Management Scheme

### 4.1. Mobility Management with Multi-Connectivity

As a UE moves in cellular systems, it periodically monitors the RSRPs from neighboring BSs. When a link from one of the neighboring BSs is offset higher than the serving link (A3 event), this link is replaced with a better link; this process is referred to as HO. The measured RSRP values are filtered at layers 1 and 3 to mitigate fading and shadowing effects, and the final HO decision is made when the A3 event is satisfied for TTT [24]. A conditional HO operation in 5G systems can improve the HO success rate by making earlier preparation decisions.

**Figure 3:**
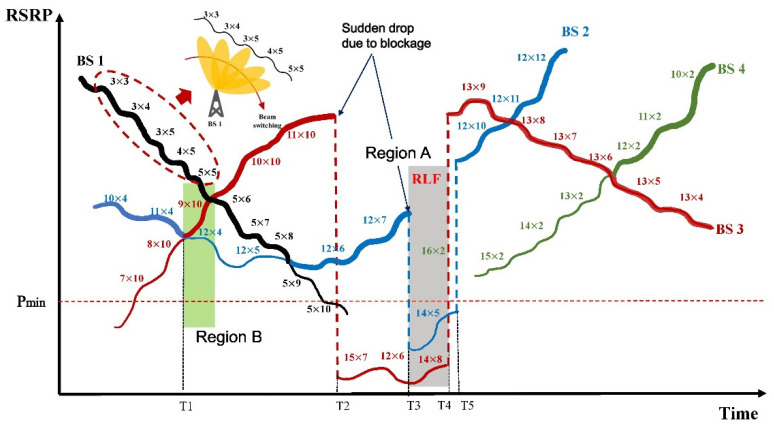
Example diagram of mobility management with MIMO (4 BSs, MC size = 2).

Figure 3 illustrates an example of mobility management; here, we have four BSs with an MC size of two. The RSRP values from the four BSs are drawn, and the thick lines indicate the serving BSs. The RSRP is measured via the optimal beam, where *h* × *v* denotes the beam index along the horizontal and vertical directions. For simplicity, the HO offset and TTT are neglected. Beam switching occurs within the same BS in addition to the HO operations between BSs. As the UE moves, the optimal beam index can be incremented or decremented by one in either the vertical or horizontal direction or in both directions simultaneously. These are the usual cases in the LoS scenario. However, in an NLoS case, the beam index change can exceed one. As urban environments can feature sudden blockages, the UE can suffer from abrupt drops and rises in the RSRP, such as at T2–T5 for BS2 and BS3. Even with multiple connections, an RLF can occur when both links are weak, as indicated in Region A. As depicted in Region B, at time T1, the RSRP from candidate BS3 becomes higher than that from candidate BS2. In this case, BS2 can be replaced by BS3 via the HO procedure to support the two best links. However, because the two existing serving BSs, BS1 and BS2, can still provide sufficient link qualities, it is not necessary to perform the HO procedure at T1. This can reduce signaling overhead and complexity while maintaining the link quality at a satisfactory level. By appropriately handling these phenomena, unnecessary HO operations can be avoided while maintaining RLF incidence at a low level.

### 4.2. Heuristic Scheme

For effective mobility management with MC in mmWave bands, a heuristic scheme is presented: an aggregated-BS (A-BS) scheme. Given the absence of any preexisting methods for executing HOs involving MC, we have formulated a heuristic scheme. This approach can be seen as a direct and uncomplicated extension of conventional HO schemes. In the A-BS scheme, an HO operation is performed when the RSRP from a candidate beam/BS is higher than the aggregated RSRP from all the serving BSs. The heuristic scheme is explained in Algorithm 1 with the offset $O_{ff}^{a}$. The number of multiple connections is denoted by the MC size, *M*. The BS or beam is replaced when a new BS/beam has a better offset quality than the aggregated or equivalently summed received power, unlike the minimum received power. The offset $O_{ff}^{a}$ is given as $O_{ff}-10\log\left(M\right)$, where $O_{ff}$ is an offset value when single connectivity is assumed, to account for the increase in power owing to the summation of the received power.

At each position, the aggregated received power of the *M* serving BSs, $P_{r,sum},$ is obtained. $B_{s}$ is a set of serving BSs and $L_{s}$ is a set of pairs $\left(b_{s}^{\left(m\right)},f_{s}^{\left(m\right)}\right)$, where $b_{s}^{\left(m\right)}$ is an m-th serving BS and $f_{s}^{\left(m\right)}$ is an serving beam of $b_{s}^{\left(m\right)}$. i.e., $L_{s}={\left\{\left(b_{s}^{\left(m\right)},f_{s}^{\left(m\right)}\right)\right\}}_{m\in \left\{1,\ldots ,M\right\}}$. Following this, for all BSs indices *b* and the beam index *f*, the received power is compared with $P_{r,sum}+O_{ff,}^{a}$, and one of the three operations, beam switching, HO, and stay, can be performed within the same BS, and HO refers to a change in the serving BS. In this scheme, the offset is adaptively set to the MC size, and the summed BS power is compared, instead of the individual BS power. This may result in reduced HOs while maintaining the required link quality in MC. Additionally, with a large MC size, the summed power tends to increase, resulting in less frequent HOs.
**Algorithm 1:** Heuristic scheme**1:** $\mathrm{Initialize}\;O_{ff}^{a}$, MC size=M$;\;L_{s}=\left\{\left(b_{s}^{\left(1\right)},f_{s}^{\left(1\right)}\right),\ldots ,\left(b_{s}^{\left(M\right)},f_{s}^{\left(M\right)}\right)\right\};$**2:** **for** Each position **do****3:**  $\mathrm{Update}\;P_{r,sum}\leftarrow P_{r}\left(b_{s}^{\left(1\right)},f_{s}^{\left(1\right)}\right)+...+P_{r}\left(b_{s}^{\left(M\right)},f_{s}^{\left(M\right)}\right);$**4:**  **for** all *b*, *f*
**do****5:**   $\;\;t=argmin\left(P_{r}\left(b_{s}^{\left(1\right)},f_{s}^{\left(1\right)}\right),\;\ldots ,\;P_{r}\left(b_{s}^{\left(M\right)},f_{s}^{\left(M\right)}\right)\right)$**6:**   $\;\mathrm{if}\;P_{r}(b,f)>P_{r,min}+O_{ff}^{a}$
**then****7:**    $\;\mathrm{if}\;\left(b,f\right)\notin L_{s}$
**then****8:**     $\;\mathrm{if}\;b\in B_{s}$
**then****9:**       $\;\mathrm{Beam}\;\mathrm{Switching}:\;\mathrm{Update}\;\left(b_{s}^{\left(t\right)},f_{s}^{\left(t\right)}\right)\leftarrow \left(b,f\right)$;**10:**          $\mathrm{Update}\;P_{r,sum}$**11:**    **else then****12:** $\;\mathrm{Handover}:\;\mathrm{Update}\;\left(b_{s}^{\left(t\right)},f_{s}^{\left(t\right)}\right)\leftarrow \left(b,f\right)$;**13:**        $\;\mathrm{Update}\;P_{r,sum}$**14:**    **end if****15:**   **else****16:**     **Stay:** Do nothing;**17:**   **end if****18:**  **end for****19:** **end for**

## 5. Multiagent Distributed Q-Learning-Based Mobility Management Scheme

### 5.1. Hierarchical Structure for an Agent

**Figure 4:**
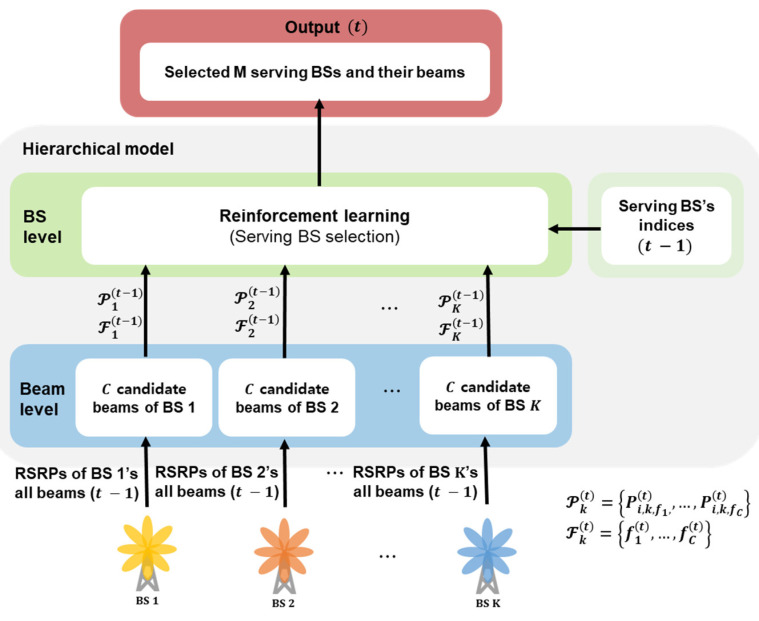
Hierarchical structure for an agent.

When a massive MIMO antenna is employed, each BS has a large number of beam indices in the horizontal and vertical directions. When we consider BS selection and beam selection simultaneously with MC, the number of possible selection combinations increases, and hence, the size of action space and the state space in Q learning models become larger. This huge size of the Q table induces poor convergence and/or memory issues. In addition, when MC is considered, the state and action sizes also increase, leading to an exponentially increasing complexity in RL. To reduce such complexity, we propose a hierarchical structure for performing mobility management separately at the beam and BS levels, as depicted in Figure 4.

At the beam level, the optimal beam is selected for each BS. The inputs here are the previous RSRP values of all beams measured from each BS. A sequence of measured RSRP values with a measurement interval of $\Delta_{m}$ can be used as an input to predict the next best beams, and various deep learning models can be adopted for this purpose [15,23]. In this study, an RSRP value averaged over *N_m_* measurement points is used as an input. This can mitigate the abrupt fluctuation of the received signal power and provide better accuracy for predicting the optimal beams and their signal powers. Given that each BS can optimally recommend the next beams, the BS level stage can focus on mobility management, including beam switching and HOs with MC and reduced complexity. The output of the beam-level stage, which corresponds to the input to the BS-level stage, is the indices of the strongest C beams and their RSRP values. Here, $P_{k}^{\left(t\right)}$ denotes the set of the RSRP values of the C recommended beams from the k-th BS at time t, and $F_{k}^{\left(t\right)}$ denotes a set of beam indices at time t. At the BS level, M optimal serving BSs are selected based on the output obtained from the beam-level stage and serving beam index at a previous point. As the UE moves along the path by one point, it selects an action based on the predicted RSRP value at each point, after which it moves to the next point. 

By using the recommended beams for each BS as input data rather than the entire beams at the BS level, the size of the Q table can be significantly decreased. In the context of full-search beam selection, the potential permutations for beam choices amount to K·NH·NVM; the number of BSs is K, the number of beams per BS is $N_{H}\cdot N_{V}$, where $N_{H}$ and $N_{V}$ are the number of antenna elements in the horizontal and vertical directions, respectively. For example, if there exist five BSs, each with 256 beams, and the MC size is two, the resulting cases sum up to 818,560. The Q table rows correspond to feasible states, while the columns denote viable action, rendering the Q table dimensions as (number of points in a route ×818,560). However, with the proposed hierarchical structure and a recommendation of C beams, the feasible permutations contract to K·CM. When C equals five, the permutations become K·CM= 300, leading to Q table size of (number of points in a route ×300). Thus, the complexity of the RL model is reduced and leads to a notable decrease in the convergence runtime of the model.

### 5.2. Distributed Multi-Agent Q-Learning (DMAQ) Scheme

Notably, multiple agents choose actions based on their own Q tables in distributed MAQ, and the outcomes are reflected in the environment shared by all agents. However, if each agent selects an action solely based on its reward and current state, it cannot consider the impacts of other agents on the environment. However, sharing channel state information or expected interference incurs an additional signaling burden, leading to a signaling overhead and time delay. Therefore, RS is proposed. In RS, local rewards are shared to reach a globally optimal reward. Consequently, every agent makes decisions on the selection of an action to achieve their own goals while avoiding disrupting one another. That is, learning proceeds by increasing the shared rewards for all agents.

**Figure 5:**
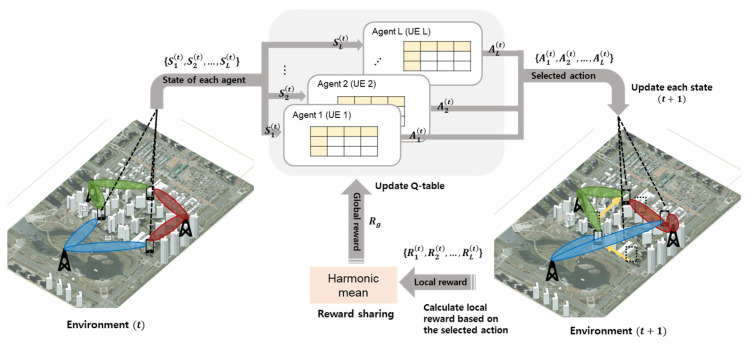
Operation diagram of DMAQ.

Figure 5 depicts the operation diagram of the DMAQ. Multiple agents are involved, and each agent updates its own Q table. Each agent selects an action based on its own state and Q table, and the actions taken by each agent are reflected in the environment. Thereafter, each agent calculates its own local reward, and finally, a shared reward or global reward is calculated at the RS stage. Every agent updates its Q table based on the global reward.

The MAQ model assumes the presence of multiple UEs in the environment. Each agent learns its own policy to achieve its own goal. The Q learning framework is similar to that of a single agent. However, in a multiagent model, several challenges are often encountered; notably, these can be avoided with the SAQ model [25]. The first challenge is that the state is nonstationary. As the environment is configured by multiple agents and affected by the choices of these multiple agents, the state that each agent receives from the environment can be nonstationary. The second challenge is the credit assignment problem. In particular, the best action for one agent can incur a penalty for another agent. Finally, while determining the policy to be used to ensure that all agents receive good rewards simultaneously, the reciprocity among agents should be considered. It is crucial to define the Q learning elements (especially the reward element) to achieve the goals of training while considering these challenges encountered during convergence in the multiagent framework scenario. 

The DMAQ model used at the BS level consists of four elements: agent, state, action, and reward.

- Agent: Each agent interacts with the environment and decides on an action based on the observed state of the environment. The UE can measure the RSRPs of all beams from the neighboring BSs. By setting the UE as an agent, the agent can employ the information collected from the measurements for learning.
- State: The state provides information to the agent regarding the current conditions of the environment. Based on this state, each agent can make a decision, and after taking an action, the state is updated to the next state. For our problem, the state consists of the M serving BS indices at the previous position and the RSRP values obtained from K BSs, including the current and K−1 neighboring BSs. The RSRP values are the outputs of the beam-level model. Hence, the state of each agent is defined as follows:
  (5) $$s_{i}=\left\{P_{1}^{\left(t-1\right)},P_{2}^{\left(t-1\right)},\ldots ,P_{K}^{\left(t-1\right)},\;\Omega_{b,i}^{\left(t-1\right)}\right\}.$$

- Action: An action of each agent is selecting the indices for the serving BSs. It decides whether the current serving BS(s) is/are to be maintained or changed. The number of possible actions for choosing M BSs from among K BSs is KM per agent.
- Reward: The reward is the feedback from the environment after taking an action. Based on this reward, the agent can evaluate its decision and learn to select the optimal action. For mobility management in this study, our goal is to reduce unnecessary HOs while maintaining an appropriate data rate. As such, the local reward of each agent is defined in terms of the achievable rate, and the HO cost $C_{HO}$ is introduced as a penalty to reduce the HOP. Using the SINR of each UE calculated in (4), the reward for the selected action is calculated as follows [26]:
  (6) $$r_{i}=\left\{\begin{array}{c} \;B\;\log_{2}\left(1+SINR_{i}\right)\;\;\;\;\;\;\;\;\;\;\;\;\;\;\;\;\;\;\;\;,\mathrm{if}\;\mathrm{no}\;\mathrm{HO}\;\mathrm{occurs}\\ B\;\log_{2}\left(1+SINR_{i}\right)\cdot \left(1-C_{HO}\right),\;\mathrm{if}\;\mathrm{HO}\;\mathrm{occurs}\;\;\;\;\;\;\;\; \end{array}\right.,$$
  where B is the bandwidth.
  Using this local reward for eachell agent, a global reward, $r_{g}$, is calculated using the harmonic mean formula to provide fairness among the users.
  (7) $$r_{g}=\frac{U}{\sum_{i=1}^{U}\frac{1}{r_{i}}}$$

- The learning algorithm of the DMAQ scheme is presented in Algorithm 2.

**Algorithm 2:** DMAQ Scheme
**1:**
 **Initialize** Q table to zero;
**2:**
 **Initialize** number of BS = K, number of UE = U, number of recommended beams C;
**3:**
 **Initialize** state S, action A, local reward $r_{i}$, shared reward $r_{g}$, total timestep number $N_{p}$;
**4:**
 **Load** RSRP data of all beams of each neighboring BS
**5:**
 **Operate** Beam-level model
**6:**
 **for** Each k
**do**
**7:**
  **Choose** The strongest C beams
**8:**
  **Save** RSRP values and indices of the chosen beams//output of the beam-level model
**9:**
 **end for**
**10:**
 **Operate** BS-level model 
**11:**
 **for** Each episode **do**
**12:**
  **for** Each timestep $N_{p}$
**do**
**13:**
   **for** all i
**do**
**14:**
    **Set** state $s_{i}$ of the UE i with the output of the beam-level model and previous    serving beam indices
**15:**
    **Select** action $a_{i}$ in state $s_{i}$ using policy derived from Q table
**16:**
    **Take** action $a_{i}$, observe the next state $s_{i}^{\prime }$, calculate local reward $r_{i}$
**17:**
    $s_{i}\leftarrow s_{i}^{\prime }$
**18:**
   **end for**
**19:**
  Calculate the global reward $r_{g}$
**20:**
   **for** all i
**do**
**21:**
    $Q_{i}\left(s_{i},a_{i}\right)\leftarrow \;Q_{i}\left(s_{i},a_{i}\right)+\alpha \left(r_{g}\left(s_{i},a_{i}\right)+\gamma \underset{a_{i}^{\prime }}{\max}Q_{i}\left(s_{i}^{\prime },a_{i}^{\prime }\right)-Q_{i}\left(s_{i},a_{i}\right)\right)$
**22:**
   **end for**
**23:**
  **end for**
**24:**
 **end for**

## 6. Simulation and Analysis

### 6.1. Channel Measurement

We first import the geographic information from the public digital elevation model provided by the National Geographic Information Institute. Subsequently, the buildings are generated based on Skyview from Naver Map. Wireless Insite provides radio wave propagation characteristics based on ray-tracing models [27]. The detailed parameters are listed in Table 1.

We obtain information on each ray in terms of path loss, signal power, AoD, direction of departure, delay, etc. Beam-formed channels are obtained by combining the ray-tracing output from Wireless Insite and appropriate post-processing with the beam-steering vectors. With horizontal and vertical departure directions of the l-th path, $\phi_{l}^{D}$ and $\theta_{l}^{D}$, respectively, the antenna element radiation pattern $A_{E}\left(\phi_{l}^{D},\theta_{l}^{D}\right)$ is adopted [29,30], where the maximum directional gain of the antenna element is 8 dBi, the front–back ratio is 30 dB, the vertical and horizontal 3 dB beamwidths are 65°, and the side-lobe level limit is 30 dB.

Then, the geometric channel H with L paths can be expressed as follows [11]:

(8) $$H=\sqrt{N_{t}N_{r}}\sum_{l=1}^{L}\alpha_{l}\alpha_{r}\left(\phi_{l}^{A},\theta_{l}^{A}\right)a_{t}^{*}\left(\phi_{l}^{D},\theta_{i}^{D}\right)\sqrt{A_{E}\left(\phi_{l}^{D},\theta_{l}^{D}\right)},$$

where $N_{t}$ and $N_{r}$ are the numbers of antennas at the transmitter and receiver, respectively, and $\alpha_{l}$ is the complex channel gain. $\alpha_{r}\left(\phi_{l}^{A},\theta_{l}^{A}\right)$ and $a_{t}^{*}\left(\phi_{l}^{D},\theta_{l}^{D}\right)$ are the steering vectors for the l-th path with the corresponding arrival and departure directions, respectively.

We perform post-processing to obtain the beam-formed channel with an $N_{H}\times N_{V}$ uniform planar array antenna at the BSs and a single antenna at the UE. The discrete Fourier transform codebook $f_{n}$ is assumed, and it is expressed as the Kronecker product of the azimuth and elevation steering vectors [31].

(9) $$f_{n}=a_{az}\left(\phi_{n},\theta_{n}\right)⨂a_{el}\left(\phi_{n}\right),$$

(10) $$a_{az}\left(\phi_{n},\theta_{n}\right)=\left[1,e^{jksin\phi_{n}cos\theta_{n}},\ldots ,e^{jk\left(\left(N_{H}-1\right)sin\phi_{n}cos\theta_{n}\right)\;}\right],$$

(11) $$a_{el}\left(\phi_{n}\right)=\left[1,\;e^{jkcos\phi_{n}\;},\ldots ,e^{jk\left(\left(N_{V}-1\right)cos\phi_{n}\right)}\right].$$

$\phi_{n}$ and $\theta_{n}$ are the steering angles of the n-th beam along the horizontal and vertical directions, respectively. Finally, we calculate the received power that UE i receives from the BS k through the n-th beam as

(12) $$P_{i,\;k,n}={\left|f_{n}*H\right|}^{2}.$$

The signal strength received via the post-processed n-th beam (https:/github.com/SiA-Ryu/WI-data, accessed on 29 August 2023) is used as the RSRP at each location of the UE. A 16 × 16 antenna configuration is adopted.

### 6.2. Simulation Results and Discussions

#### 6.2.1. A Single UE Case

As a UE moves to a point, it obtains the received signal power from all the possible beams. HO or beam switching is performed according to the heuristic scheme or single agent Q learning (SAQ) scheme. The SAQ algorithm is a single UE case of the proposed DMAQ. The offset value $O_{ff}$ of the heuristic scheme is set to 2 dB.

**Figure 6:**
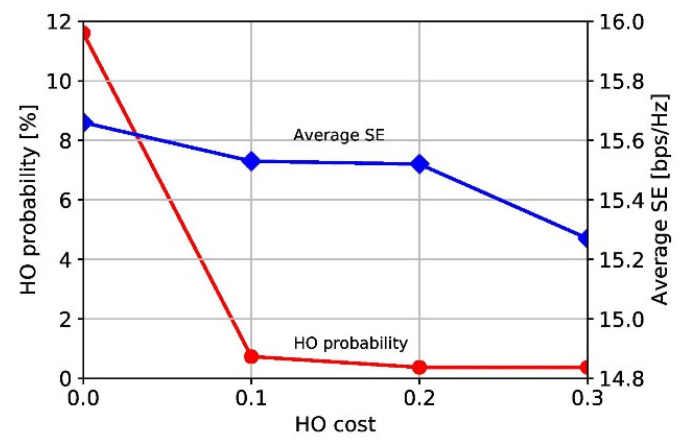
Average SE and HOP in SAQ with different HO costs.

We adopt SE and HOP as pivotal performance metrics. The SE quantifies data transmission capacity and HOP offers valuable insights into the stability and efficiency in mmWave cellular networks. We first investigate the impact of the HO cost by varying the value in the range of 0 to 0.3 with an MC size of two. As expected, as the HO cost increases, the HO probability (HOP) and SE decrease, as shown in Figure 6. With a high HO cost, the HO operation is delayed as much as possible, thereby reducing the HOP while degrading the SE. In the subsequent simulations, the HO cost is set to 0.2.

The average SE and HOP of the heuristic scheme and SAQ scheme are summarized with the MC size of 1 and 2 in Table 2. Compared to the heuristic scheme, the HOP performance can be greatly improved with slight degradation in average SE performance in the SAQ scheme because the number of unnecessary HO operations can be further reduced. Additionally, as the MC size increases from 1 to 2 in each scheme, the average SE increases and unnecessary HO is reduced because the UE determines HO based on the aggregated RSRPs of the two BSs. If the RSRP of the one connection is good enough, unnecessary HO does not occur.

#### 6.2.2. Two UEs Case

We assume that two UEs use the same resource. An independent Q learning (IQL) scheme is additionally considered as a foundational model for multi-agent scenarios, maintaining autonomy among agents and excluding RS.

The performance is evaluated in terms of the average SE and HOP for MC sizes of two in Table 3. Compared to the heuristic scheme and the IQL scheme, the average SE of the DMAQ scheme is significantly improved. When RS is applied, a better BS and beam can be selected to achieve a larger global reward considering interference from another UE’s serving beam, and hence the sum SE performance can be further enhanced. Compared to heuristic and IQL schemes with an MC size of 1, the average SE performance of the proposed DMAQ scheme increased 7.2% and 13.1%, respectively. On the other hand, the HOP increases in the DMAQ scheme. This is because the serving BSs or beams are selected in consideration of the dynamically fluctuating interference originating from neighboring BSs whose beams serve the other UE. The selection of the BS and its beam must be performed to increase the RSRP and minimize interference with other UEs at the same time. However, this makes BS/beam selection in the DMAQ scheme more complex and invokes more frequent HOs, which, in turn, improves the SE performance. When the MC size is increased to two, HO operations occur more frequently than when the MC size is one. This is different from the single UE case, in which no interference originates from neighboring BSs. Compared to the single UE case, the HOP is relatively high in DMAQ. This is because switching BS or beam indices occurs more frequently than maximizing serving signal strength while minimizing interference to other UE. In the IQL scheme and heuristic scheme, the HOP is as low as in the single UE case because each UE determines serving BS/beam without considering the interference to other UE; however, the average SE performance is degraded.

**Figure 7:**
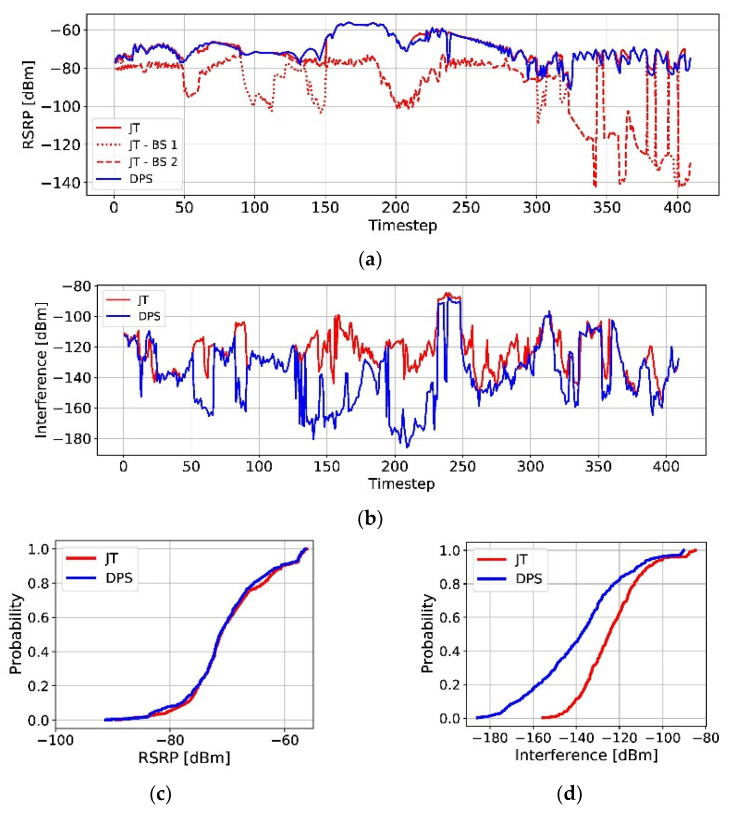
Comparison of JT with DPS in DMAQ: (**a**) RSRP of each serving BS over the timesteps at UE1; (**b**) Interference over the timesteps; (**c**) RSRP CDF; (**d**) interference CDF.

The SE and HOP values in DPS and JT transmission modes are summarized at the bottom of Table 3. In particular, DPS mode can provide slightly better SE performance with a reduced HOP. Figure 7a depicts traces of the RSRP values for UE 1 for JT and DPS. For JT, a relatively weak connection is maintained because the MC size is fixed at two. However, by applying the DPS transmission mode and preserving a single strong connection, the RSRP can be maintained at a sufficiently high level compared to that in JT mode, as shown in Figure 7c. Notably, interference can be significantly reduced by DPS mode, as shown in Figure 7b,d. Because only one beam is used for transmission in DPS mode, the impact of the interference on the other UE is significantly reduced, so the average SE in DPS mode is larger than that in JT mode. Further, in our analysis, a real map environment with numerous buildings is considered, and a 16 × 16 antenna configuration is used in the simulation. The beams are very sharp with continuous blockages (such as buildings). Additionally, via the RS concept, the beam with predominant interference can be excluded in the selection stage. Hence, interference can be controlled to remain as low as possible.

#### 6.2.3. Impact of MC Size

**Figure 8:**
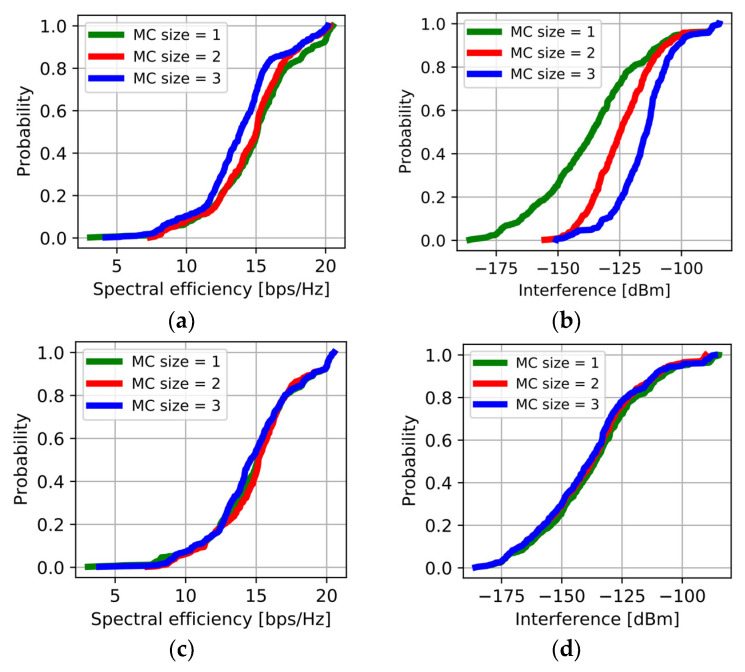
Comparison by the MC size in DMAQ: (**a**) SE in JT mode; (**b**) Interference in JT mode; (**c**) SE in DPS mode; (**d**) Interference in DPS mode.

The impact of MC size on the performance of JT and DPS transmission modes are investigated. Comparing the two modes, JT and DPS, the cumulative distribution functions (CDFs) of SE and interference are examined with respect to different MC sizes. Figure 8a,b illustrates the findings for JT mode. Surprisingly, SE performance reaches its peak at an MC size of 1 but deteriorates as the MC size increases to 3. In JT mode, increasing the MC size results in the summing of received signal strengths from multiple beams used for transmission. However, Figure 8b shows that as the MC size increases, so does the interference from other UE serving beams. The impact of increased interference outweighs the gain in received power, leading to a degradation in SE. Turning to DPS mode, Figure 8c,d present the CDFs of SE and interference performance. Notably, in DPS mode, even with larger MC sizes, only one beam is utilized for transmission, keeping the interference at a nearly constant level. Remarkably, Figure 8d demonstrates that compared to JT mode with an MC size of 3, the interference is reduced by approximately 20% in DPS mode. In the specific urban scenario considered, the received signal strength of the second strongest beam is relatively lower than that of the strongest link. Consequently, the gain in signal strength as the MC size increases is not significant. However, in JT mode, the interference experiences a substantial increase, resulting in a degradation of SE. This phenomenon is not observed in DPS mode, where interference is effectively mitigated. Overall, the findings indicate that, in the given urban scenario, increasing the MC size beyond 1 in JT mode does not yield notable benefits in terms of SE due to the significant increase in interference. In contrast, DPS mode maintains a consistent interference level, resulting in improved SE performance compared to JT mode with larger MC sizes.

Table 4 provides insight into the impact of MC size on the HOP in JT and DPS modes. The trends observed in HOP performance differ significantly between the two modes as the MC size increases. In JT mode, the HOP exhibits distinct behavior with varying MC sizes. When the MC size is 2, the HOP increases compared to the case with an MC size of 1. As the MC size increases to 3, the HOP decreases slightly but remains higher than when MC size is 1. The HOP is calculated by counting the number of changed BS indices when either the serving or transmitting beams are switched to beams from another BS. With an MC size of 2, there are two possible beams that can be changed and counted as an HO, resulting in an increased HOP. When the MC size is increased from 2 to 3, the received signal strength of the third strongest beam is significantly lower than the others, leading to similar HOP performance. Conversely, in DPS mode, the HOP decreases as the MC size increases. Since only one of the connected beams is used for transmission, changing the beam index is not considered an HO when one of the connected beams is switched to a transmitting beam. Additionally, reward calculation is based on a single transmitting beam. Thus, if the received signal strength of the transmitting beam is sufficiently high to ensure good SE performance, there is no need to change the other connected beams. Consequently, the HOP is relatively lower compared to JT mode, and unnecessary HOs can be avoided with the MC provided by DPS mode. This indicates that DPS mode is particularly beneficial for MC scenarios in mmWAVE cellular systems. Overall, the results from Table 4 demonstrate the contrasting trends in HOP performance between JT and DPS modes as the MC size increases. While JT mode shows an increase or marginal decrease in HOP, DPS mode exhibits a consistent reduction in HOP, highlighting its advantages for MC scenarios in mmWAVE cellular systems.

#### 6.2.4. Impact of the Number of UEs

**Figure 9:**
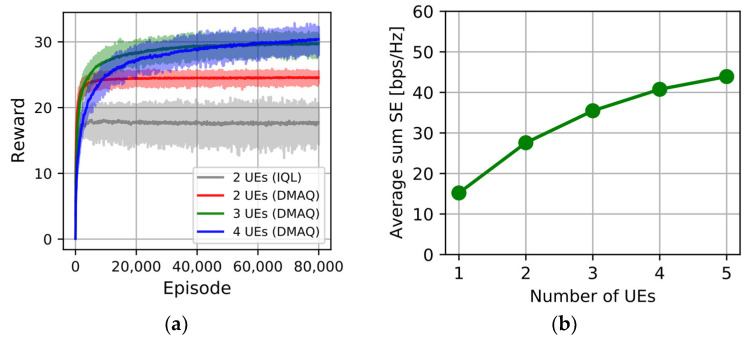
DMAQ with different numbers of UEs: (**a**) reward convergence; (**b**) average sum SE.

Figure 9a shows the reward convergence of the IQL algorithm and proposed DMAQ algorithm by varying the number of UEs with an MC size of 2. It takes more episodes for the Q table to converge, since the size of the Q table becomes larger as the number of UEs increases. In the case of the IQL scheme, since the interference between UEs cannot be considered, the converged reward is significantly lower compared to the DMAQ schemes. The average sum SE performance is shown in Figure 9b. As the number of UEs increases, the average sum SE increases but the amount of SE increase decreases, i.e., the sum SE does not increase linearly with the number of UEs. As more UEs are serviced simultaneously, the interference increases, which results in decreased SINR, and accordingly, the individual SE is decreased. However, the sum SE is the multiplication of the number of UEs and the individual SE, and hence, even though the individual SE decreases, the sum SE can increase, but the gain decreases as the number of UEs increases. In addition, the HOP also increases as the number of UEs increases. This is because the interference from multiple UEs may invoke more frequent reselection of beam and BS to achieve optimal reward in the DMAQ model.

## 7. Conclusions

We proposed a DMAQ-based mobility management scheme for the support of MC in mmWave cellular systems. A hierarchical structure was adopted to mitigate the model complexity and convergence problem, and RS was proposed for a distributed MAQ model to obtain global optimal performance with minimal overhead. The performance was evaluated based on a realistic measurement data set obtained from Wireless Insite and real map information. The heuristic scheme and the SAQ model could provide similar SE values, although the SAQ model resulted in a lower HOP. The DMAQ scheme could significantly improve the SE by simultaneously servicing multiple UEs with the lowest interference possible owing to global optimization with RS. In addition, we compared JT mode and DPS mode. When DPS mode was applied, the influence of unnecessary interference was reduced and the SE performance was improved. The unnecessary number of HOs could be reduced while maintaining a sufficient level of SE by utilizing the MC of DPS mode.

The proposed algorithm can be successfully employed in practical UEs with limited computation power and battery life since the DMAQ does not require a deep neural network for function approximation. For further work, to enable the exploration of more complicated and pragmatic scenarios, we will apply the deep RL framework to our proposed model. Addressing the challenge of restricted computation power or battery life of UEs can be facilitated through the use of BS or edge computing technology. Also, mobility management faces new challenges in future networks including vehicle-to-everything (V2X), air-to-everything (A2X), HetNets, mobile edge computing (MEC), and tera-Hertz band, where ML plays a more powerful role to consider complex factors such as new channel models, service requirements, combination with localization and sensing information and so on.

## Figures and Tables

**Table 1:** Wireless Insite parameters.

Parameter Description	Value
Carrier frequency	28 GHz
Frequency bandwidth [28]	100 MHz
Transmitted power	34 dBm
Propagation model	X3D
City material	Concrete
Ray spacing	0.33
Number of reflections	6
UE speed	30 km/h

**Table 2:** Average SE and HOP for a single UE case.

Scheme	MC Size	Average SE [bps/Hz]	HOP [%]
Heuristic (A-BS)	1	15.42	1.94
	2	15.69	1.47
SAQ	1	15.2	0.61
	2	15.53	0.49

**Table 3:** Average SE and HOP for two UEs case.

Scheme		MC Size	Average SE [bps/Hz]	HOP [%]
Heuristic (A-BS)		1	12.9	1.58
		2	11.56	0.85
IQL		1	12.09	0.73
		2	12.24	0.49
DMAQ	JT	1	13.91	3.7
		2	13.79	4.77
	DPS	1	13.91	3.7
		2	14.03	2.92

**Table 4:** HOP for JT and DPS modes according to MC size.

MC Mode	JT	DPS
MC Size		
1	3.7%	3.7%
2	4.77%	2.92%
3	4.16%	1.46%

## Data Availability

Not applicable.
